# Ultra-fast Hygrometer based on U-shaped Optical Microfiber with Nanoporous Polyelectrolyte Coating

**DOI:** 10.1038/s41598-017-08562-1

**Published:** 2017-08-11

**Authors:** George Y. Chen, Xuan Wu, Yvonne Qiongyue Kang, Li Yu, Tanya M. Monro, David G. Lancaster, Xiaokong Liu, Haolan Xu

**Affiliations:** 1 Laser Physics and Photonic Devices Laboratories, School of Engineering, University of South Australia, Mawson Lakes, South Australia 5095 Australia; 2 Future Industries Institute, University of South Australia, Mawson Lakes, South Australia 5095 Australia; 3 Shenzhen Key Laboratory of Laser Engineering, College of Optoelectronic Engineering, Shenzhen University, Shenzhen, 518060 China; 4 Key Laboratory of Optoelectronic Devices and Systems of Ministry of Education and Guangdong Province, College of Optoelectronic Engineering, Shenzhen University, Shenzhen, 518060 China

## Abstract

Real-time measurement of the relative humidity of air has applications ranging from process control to safety. By using a microfiber form-factor, we demonstrate a miniature and fast-response hygrometer with the shortest-ever response time (3 ms). The sensor head consists of an optical microfiber of 10 µm diameter and 2 mm length configured to form a compact U-shaped probe, and functionalized with a polyelectrolyte multilayer coating of 1.0 bilayer. The sensing mechanism is primarily water-absorption-based optical loss. We have measured a response time of 3 ms and a recovery time of 36 ms. The sensitivity is as high as 0.4%/%RH, and the detection limit is as low as 1.6%RH. The maximum relative humidity is 99%RH, before reaching a recoverable dew-point.

## Introduction

The measurement and control of humidity is imperative for environments including the atmosphere, agricultural (e.g. greenhouses, crop fields), buildings (e.g. museums, heritage buildings, operating theatres, rehabilitation wards), food (e.g. baking, drying, storage), medical (e.g. smart bandages, diagnostic tools) and manufacturing (e.g. glasses, coatings, fibers). Electrical humidity sensors, also known as hygrometers, based on resistivity or capacitance changes are well established^1–4^. Electrical hygrometers are unsuitable for applications requiring the sensors to be compact and lightweight for good portability, flexible enough to probe in and retract from confined spaces, immune to electromagnetic interference (EMI), completely inert within combustive environments, and sufficiently robust to last a few years. Driven by these demands, fiber-optic hygrometers have emerged^5–12^, and they typically have response times ranging from tens of milliseconds to seconds. Existing hygrometers must be faster for applications that require faster temporal response, such as: (a) process control for mass production of chemical compounds^13,14^, where humidity is regulated as a part of quality assurance; (b) touchless keypads for information systems, where selectiveness to bare skin leads to less false triggers than capacitive/resistive-touch technologies; (c) water-hazard safety mechanisms for portable electronics (e.g. laptop computers) and undersea environments (e.g. Eurotunnel); (d) respiratory analyzers for identifying illnesses, where water vapor is less hampered by noise compared to air flow; and (e) atmosphere mapping for more accurate weather forecasts, where high spatial-resolution mapping of the atmospheric relative humidity (RH) via unmanned aerial vehicles can improve weather simulation models. Addressing this need can: (i) increase productivity; (ii) promote the safety of undersea personnel; and (iii) unveil information that can potentially benefit health. The best performances so far have been 30 ms^5^ (i.e. optical, nanowire) and 8 ms^1^ (i.e. electrical, nanofiber) response times. Although the latter is considerably faster, it is not immune to EMI. The main challenge to date has been finding a material and sensor design that can achieve millisecond-scale temporal responses, while exhibiting the aforementioned traits.

**Figure 1:**
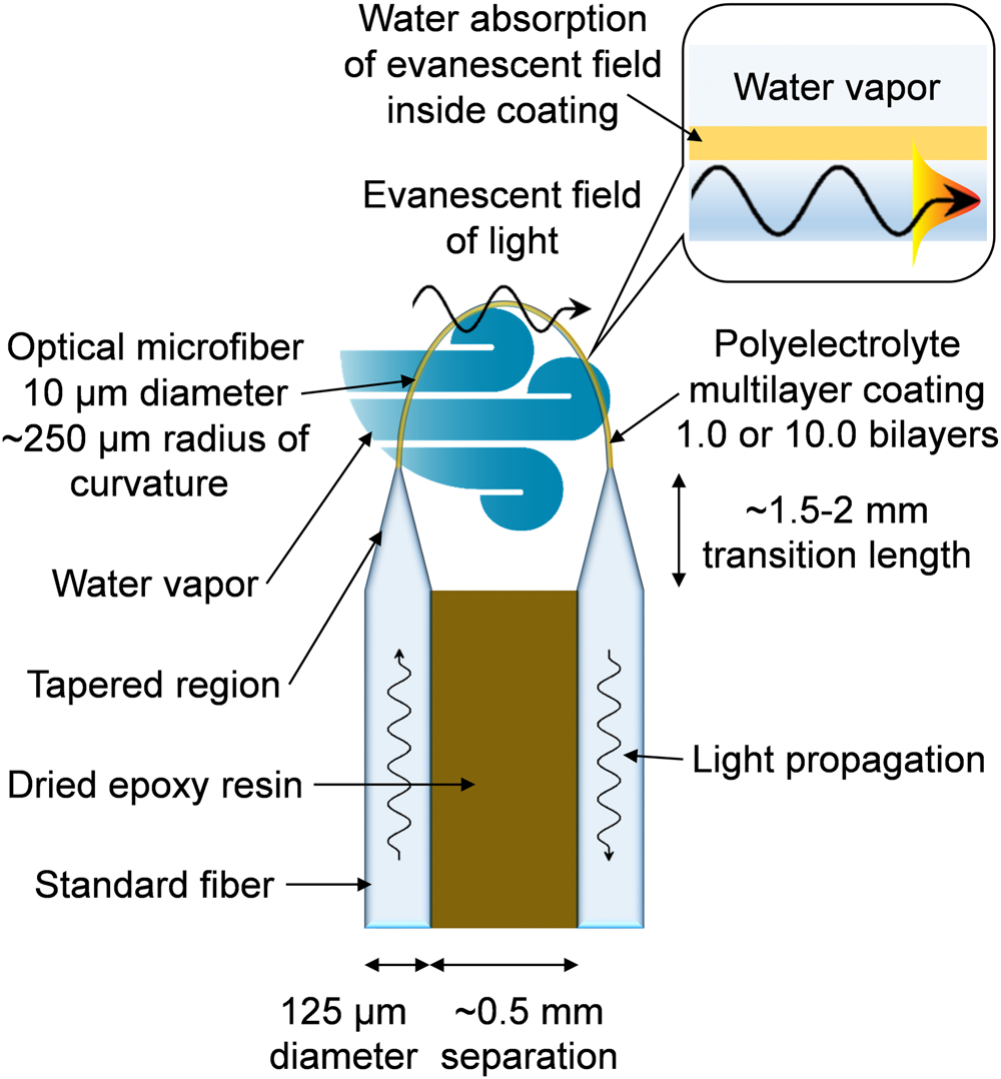
Schematic of the sensor head. Inset: side-view structure of sensor head with the optical microfiber at the bottom and external environment at the top.

Recently, layer-by-layer-assembled polyelectrolyte multilayer (PEM) coatings featuring nanoporous-based hydrophilicity have been optically interrogated on silicon substrates for humidity-based touchless control, exhibiting a fast response time of 35 ms and wavelength shift of the reflectance spectrum exceeding 305 nm for 0–99%RH^12^ (i.e. 6 × 10^−3^%RH detection limit for 20 pm wavelength resolution). The coating displays vibrant colors via thin-film interference, for a range of coating thicknesses in the order of ~100 nm. We report here the fastest-ever hygrometer consisting of an optical microfiber of 10 µm diameter and 2 mm length configured to form a U-shaped probe, and functionalized with a single-bilayer PEM coating shown in Fig. 1. The achieved response time of 3 ms is one order of magnitude shorter than the optical record of 30 ms^5^, and shorter than the electrical record of 8 ms^1^, attributed to the combination of thin coating and circular geometry. It harnesses the excellent optical (e.g. evanescent field of light, immunity against EMI) and mechanical qualities (e.g. minimum bend radius, can operate within confined spaces with its flexible fiber connections) of optical microfibers, and the impressive absorption/desorption qualities of thin PEM coatings. The optical sensor head has the potential to be manufactured at a low cost.

## Results and Discussion

### Fabrication

The primary motivation of this experiment is to demonstrate a fast response time by reducing the coating thickness. The secondary motivation, outside the scope of this work, is to achieve a high sensitivity, by reducing the fiber taper diameter.

The first stage is to fabricate optical fiber tapers from silica glass fibers. The starting optical fiber (i.e. Nufern GF1) has coating/cladding/core diameters of 250/125/9 μm. Tapering was performed by a fusion splicer (i.e. Fujikura FSM-100P+). The sensor head starts with the uniform-waist section of a biconical fiber taper, known as an optical microfiber. Optical microfibers are widely used for sensing^15–18^, due to their excellent optical (e.g. evanescent field of light, immunity against EMI) and mechanical qualities (e.g. minimum bend radius). For a typical polymer-cladded microfiber tapered from standard telecom single-mode fiber, more than 50% of the optical power resides in the evanescent field outside the optical microfiber when its diameter is less than the wavelength of probing light^18^. This is because the tapered core has either diffused into the cladding or it remains but no longer exert much influence on mode guidance. Hence, the guided light leaks out to the cladding-surrounding interface, where its evanescent field interacts with the humidity-sensitive coating.

To deliver a balance between (a) sensitivity; and (b) robustness, turbulence resistance and coating uniformity; a microfiber diameter of 10 μm was chosen for the sensor heads. The uniformity of the microfiber diameter can be ensured in several ways. If a fusion splicer is employed, its predetermined algorithm involving differential motor velocity and arc power will ensure a uniform waist. If a custom-built tapering rig is used, either a predetermined algorithm or real-time video processing for diameter correction must be used. For each sensor head, the optical microfiber was bent into a U-shape with a bend radius of ~250 μm. Then, it was packaged by ultraviolet-light curable epoxy resin along the parallel section of standard optical fiber, leaving the tapered section exposed. When the fiber is bent, light leaks out from the single-mode core into the cladding, where it is partially guided within multiple modes. This allows the evanescent field to extend into the coating, thus creating stronger interaction of the light with the PEM coating, and thus enhanced sensing performance. The fiber tapers are not longitudinally symmetrical, due to the single-sweep method of the fusion splicer. The U-shaped probe consists of one taper transition being closer to the middle of the bend than the other side. This is a side effect from enhancing the evanescent field for sensing, by pulling on one end to reduce the bend radius.

The second stage is to deposit humidity-sensitive PEM coatings onto the optical microfibers. It consists of poly(diallyldimethylammonium) (PDDA+)/poly(styrenesulfonate) (PSS-) multilayers, which were prepared via alternate dip coating of PDDA and PSS on optical fiber tapers. The resultant PEM coatings are denoted as (PDDA/PSS)_*N*_ where *N* represents the number of deposited bilayers. A half and whole bilayer denote the coatings with PDDA and PSS as the outmost layer, respectively. The coating thickness for PDDA/PSS has been known to increase exponentially with the number of bilayers^12^, attributed to polyelectrolyte diffusion^19^. Although described as a multilayer, a PEM coating can be treated as an optically homogeneous layer due to: (a) negligible refractive-index variation across the coating due to polyelectrolyte diffusion; (b) sub-wavelength thickness of the individual layers; and (c) sub-wavelength diameter of the nanopores. The response of a PEM coating to an increase in the local humidity is water adsorption then diffusion through their nanopores followed by swelling, resulting in an increase in water-absorption-induced optical loss, increase in coating thickness, and decrease in refractive index. In comparison, an uncoated pristine microfiber is unresponsive to water vapor below 100%RH (i.e. dew point), because it is hydrophobic unless electrostatically charged. An uncoated piranha-solution-treated microfiber exhibits much lower sensitivity, due a smaller overlap between the evanescent field of the optical microfiber and the thinner layer of water molecules. In addition, it has a slower response (i.e. 10 ms), due to a weaker hygroscopic level. Both of these designs are inferior to the coated microfiber, and their performances were experimentally verified.

**Figure 2:**
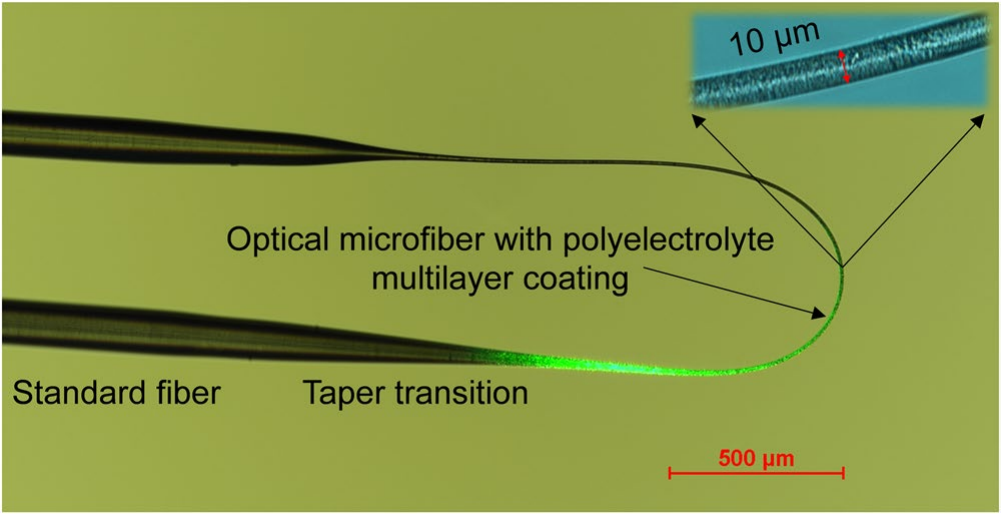
Microscope image of the sensor head, consisting of a U-shaped microfiber functionalized with polyelectrolyte multilayer coating. Green light was launched into one end of the optical microfiber to give an insight into the evanescent field of light.

To demonstrate that a thinner coating exhibits a faster response, coating thicknesses of 1.0 bilayer (i.e. 55 ± 50 nm) and 10.0 bilayers (i.e. 1190 ± 50 nm) were assembled on two optical microfibers for a comparison study. Whole bilayers with PSS as the outer layer were chosen because they exhibit a rougher porous morphology^12^ that expedites absorption/desorption of water molecules. A 0.5 bilayer microfiber was found to be impractical even though it is thinner (i.e. potentially faster), because it has poorer sensitivity (i.e. indistinguishable from noise) from its lower water-holding capacity (i.e. lower water-absorption-induced optical loss) and a smoother surface (i.e. lower hydroscopic-level) from PDDA as the outlayer. The coating thicknesses were measured and a nanoporous surface morphology was revealed using a scanning electron microscope (SEM), as shown in Fig. S1. A packaged sensor head is shown in Fig. 2. The measured optical losses were 6.7 dB and 7.5 dB respectively, due to bend- and coating-induced losses.

### Sensitivity and detection-limit measurements

**Figure 3:**
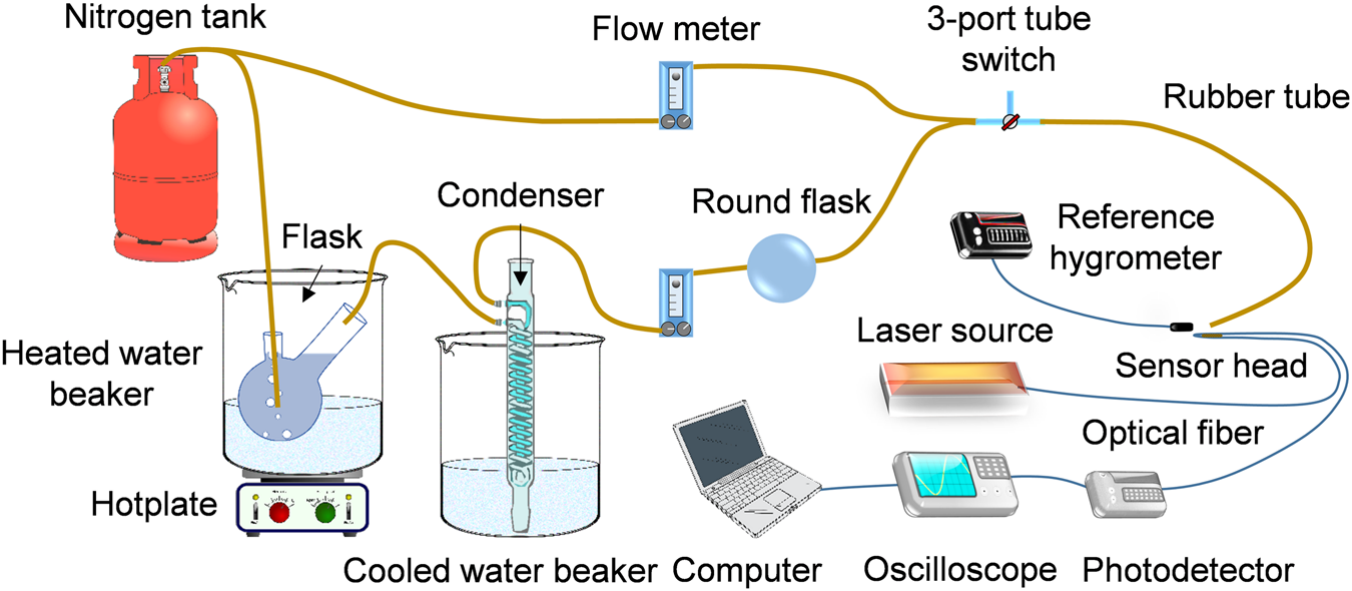
Schematic of the interrogation setup. Wet and dry nitrogen gas are injected through a series of components before being directed onto the sensor head. The optical transmission signal is converted into an electrical signal for data capture.

To measure the sensitivity of each sensor head shown in Fig. 3, linearly polarized single-mode light of 1550 nm wavelength from a diode-laser source (i.e. New Focus TLB-6728-P-D) was launched into standard optical fiber (i.e. Nufern GF1). The standard fiber was fusion spliced to one end of the coated microfiber, with the other end spliced to another section of standard fiber that fed into a photodetector (i.e. Thorlabs PBD450C, 10^3^ transimpedance gain, 150 MHz bandwidth). A sampling oscilloscope (i.e. Picoscope 6404 C) converted the analogue signal into digital data that was displayed on a computer. A moving average equivalent of an integration time of 0.8 ms was employed (e.g. 16-point filter for 200,000 points over 10 s).

The PEM coating on the optical microfiber under test was exposed to different levels of RH. The nitrogen gas was controlled by mixing dry and wet nitrogen gas. The wet nitrogen gas was generated by passing dry nitrogen gas through 70 °C and 21 °C water-filled beakers followed by a condenser to remove excess water. The mixing ratio was regulated via two flow meters, which yield a total flow rate of 5.0 litres per minute (LPM). The insertion of a 3-port tube switch before the output ensured that the system pressure was kept constant despite switching between the ambient and the output RH. The output RH was determined using a commercial hygrometer (i.e. RisePro, 0.5%RH accuracy) placed in front of the tube exit. The sensor head was interrogated by blowing nitrogen gas through a 5 mm diameter plastic tube positioned at 15 mm from the sensor head. To minimize the impact of air flow on the geometrical stability of the sensor head, the directionality was made as co-axial as possible towards the tapered section. For measuring the sensitivity, the switch is configured as straight-through. For better RH accuracy and stability, the sensor head with the delivery tube end was placed inside a simple transparent enclosure.

**Figure 4:**
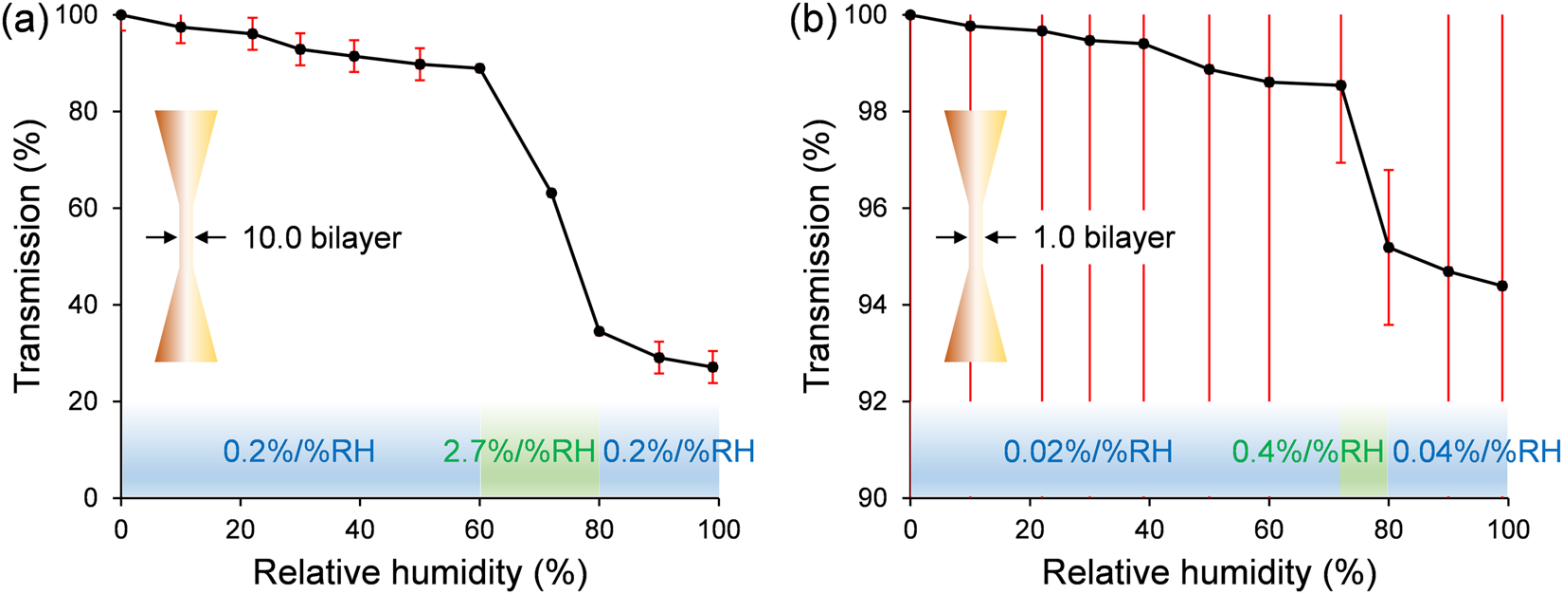
Optical transmission as a function of RH for: (**a**) 10.0 bilayer, and (**b**) 1.0 bilayer microfibers.

To determine the sensitivity involves plotting the optical transmission (i.e. voltage normalized against maximum voltage) as a function of the known RH, and finding a gradient(s) shown in Fig. 4. For the 10.0 bilayer microfiber, the sensitivity was measured to be 0.2%/%RH (i.e. 0–60%RH, 80–99%RH), and 2.7%/%RH (i.e. 60–80%RH). For the 1.0 bilayer microfiber, the sensitivity is 0.02%/%RH (i.e. 0–72%RH), 0.4%/%RH (i.e. 72–80%RH), and 0.04%/%RH (i.e. 80–99%RH). The 10.0 bilayer microfiber has a higher humidity-sensitivity than the 1.0 bilayer microfiber. This is expected because the penetration depth of the evanescent field^20^ of the cladding-guided rays into the effectively homogeneous PEM coating under ambient conditions is at least 481 nm (i.e. see Equation 1). As a result, the overlap (i.e. >481 nm and <1190 ± 50 nm) between the evanescent field (i.e. >481 nm) and the 10.0 bilayer PEM coating (i.e. 1190 ± 50 nm coating thickness) is significantly larger than the overlap (i.e. 55 ± 50 nm) between the evanescent field (i.e. >481 nm) and the 1.0 bilayer PEM coating (i.e. 55 ± 50 nm coating thickness).

1 $$\Psi =\frac{\lambda }{2\pi \cdot \sqrt{{n}_{fiber}^{2}\,{\sin }^{2}\theta -{n}_{coating}^{2}}}$$

where *λ* is the wavelength of light, *n*_*fiber*_ = 1.444 is the refractive index of the silica microfiber, *n*_*coating*_ = 1.35 is the refractive index of the PEM coating, and *θ* is the incident angle^20^ between the ray(s) and the normal to the fiber-coating interface (i.e. *θ* = 90.0° for the shallowest penetration depth).

The inverse relationship between optical transmission and RH in Fig. 4 is mainly attributed to the increasing RH-induced increase in water-absorption-induced optical loss of 1550 nm wavelength light^21^. The weaker counteracting effects with increasing RH are: (a) coating swelling^12^ leading to a larger bend radius, leading to a decrease in bend-induced loss, but suppressed by the significantly larger volume of optical microfiber bonded to the coating; and (b) decrease in refractive index^12^ leading to an increase in optical confinement, leading to a decrease in optical leakage; but relatively insensitive judging by the increasing optical loss with increasing RH. These two effects contribute to the slowdown of the drop in optical transmission beyond 80%RH. The optical response was found to be the same regardless of the direction of light propagation, because the straight fiber-taper profile maintains a single mode, and thus high-order mode excitation (i.e. evanescent field extension) and leakage only occurs at the bent region of the optical microfiber.

The noise amplitude was measured to be 19.5 mV for a signal level of 2990.0 mV over a period of 3 s, which also reflects the repeatability of the measurements across 0–99%RH. This yields a system noise of 0.652% (i.e. transmission). The accuracy of the reference hygrometer and the system noise are used for the X and Y error bars of Fig. 4 respectively. The detection limit and accuracy (i.e. error bars of Fig. 4) are calculated by dividing the system noise by the sensitivity. For the 10.0 bilayer microfiber, the detection limit and accuracy are 3.3%RH (i.e. 0–60%RH, 80–99%RH) and 0.2%RH (i.e. 60–80%RH). For the 1.0 bilayer microfiber, the detection limit and accuracy were measured to be 32.6%RH (i.e. 0–72%RH), 1.6%RH (i.e. 72–80%RH) and 16.3%RH (i.e. 80–99%RH). The detection limit and accuracy of the 10.0 bilayer microfiber is better than that of the 1.0 bilayer microfiber. The large error bars of Fig. 4(b) are caused by the low sensitivity of the 1.0 bilayer microfiber. These do not affect the actual response time (i.e. related to humidity rather than system noise) nor the observed response time (i.e. fluctuations are usually not visible in short-duration slopes, even if they are they can be removed with image-processing techniques that do not distort the signal), but the detection limit and accuracy of the sensor. The detection limit and accuracy can be improved by using a laser source with a higher optical-power output (e.g. >tens of milliwatts) and/or an oscilloscope with a lower noise-floor. The dynamic range for both sensor heads is 0–99%RH.

### Temporal characteristics measurements

**Figure 5:**
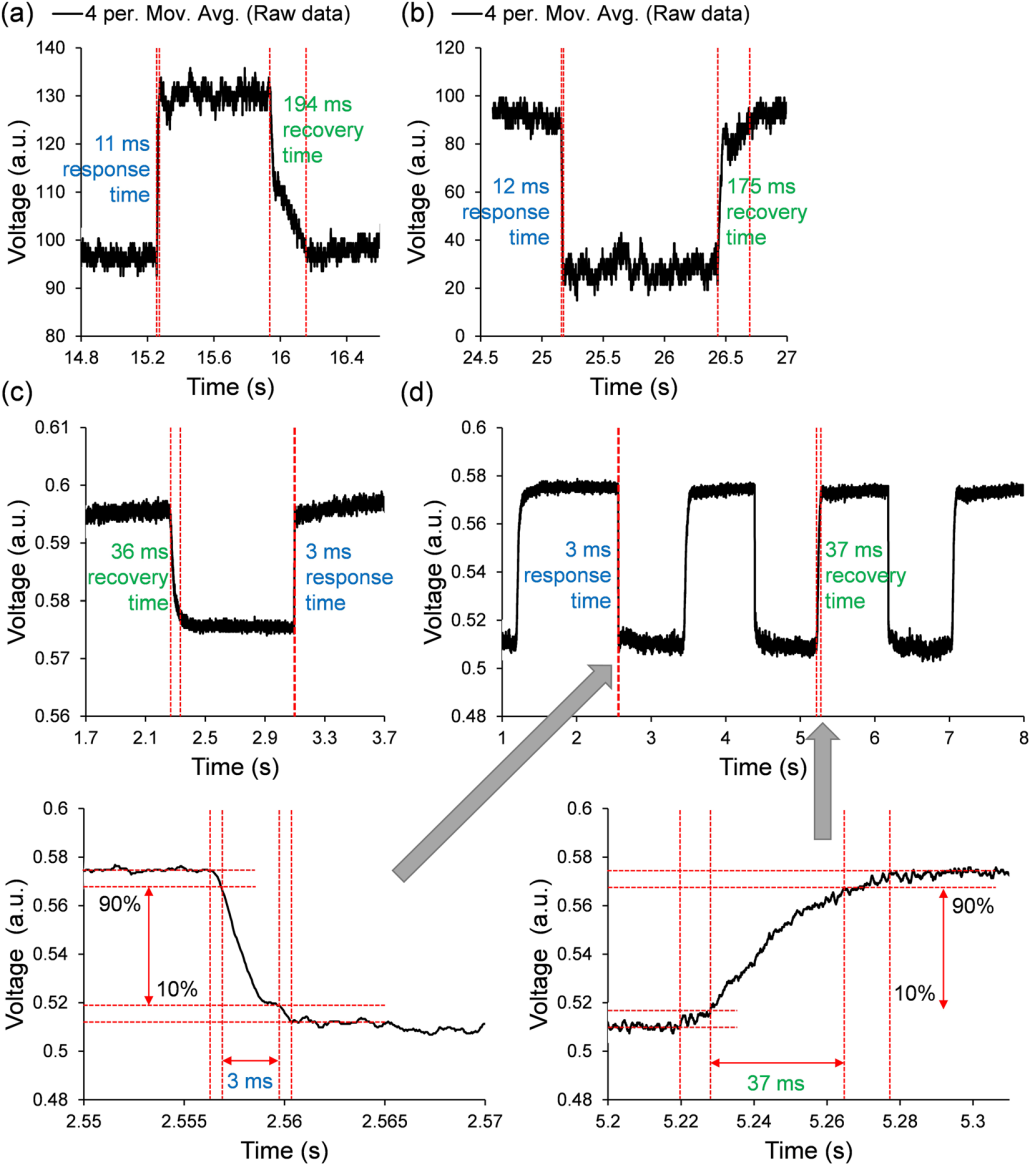
Temporal characteristics for: (**a**) 10.0 bilayer: 50–0–50%RH; (**b**) 10.0 bilayer: 50–99–50%RH; (**c**) 1.0 bilayer: 0–50–0%RH; and (**d**) 1.0 bilayer: 99–50–99%RH.

To measure the temporal response of each sensor head shown in Fig. 5, the experimental setup from Fig. 3 was reused, but without an enclosure around the sensor head to ensure free air movement. Nitrogen gas of 0%RH and 99%RH for the 10.0 bilayer and 1.0 bilayer microfibers respectively were applied through the rubber tube. The standard approach to suppress equilibrium-ambiguity was adopted by taking the temporal characteristics between 10–90% of the maximum voltage. For the 10.0 bilayer microfiber, the response and recovery times were measured to be 11–12 ms and 175–194 ms respectively. For the 1.0 bilayer microfiber, the response and recovery times were measured to be 3 ms and 36–37 ms respectively. As anticipated, the response time of the sensor head with the thinner coating is shorter than that of the thicker coating, and beats the optical record of 30 ms^5^ by one order of magnitude as well as the electrical record of 8 ms^1^. It was found that changing the RH does not visibly affect the temporal characteristics. The repeatability of the probes are typically within ± 2 ms for response times and ± 20 ms for recovery times. The variations are mainly caused by the inconsistent human hand at manually switching the 3-port tube. The consistency can be increased if a chopper is used.

We hypothesize that the extremely short temporal characteristics exhibited by the PEM coating are attributed to: (a) the nanoporous-based hydrophilic nature of both PDDA and PSS components; (b) the strong electrostatic interactions between PDDA and PSS chains; (c) the nanoscale coating thickness; and (d) the 360° outward-facing exposure angle of the coating, which facilitates a more or less uniform wavefront of moisture propagation, such that the response time incurred at the light-water interaction stage is minimized (i.e. not governed by the water-vapor propagation stage). The first and third factors enhance moisture diffusion through the coating. The second factor enables swift spreading of localized swelling or shrinking of the coating. The fourth factor facilitates rapid absorption or desorption of water molecules. The fabricated optical microfibers feature faster temporal characteristics than the previously reported planar substrates^12^, due to factors (c) and (d). The magnitude of the optical response is related to the overlap between the evanescent field and the PEM coating. Hence, a thinner PEM coating around an optical microfiber results in faster response/recovery times, but lower sensitivity. A PEM coating around a narrower optical microfiber yields higher sensitivity^22^, but it is less robust, less turbulence resistant and less likely to host a uniform coating.

### Additional measurements

The impact of flow rate on the geometrical stability of the sensor head was found to be negligible, by blowing nitrogen gas of the ambient RH (e.g. 50%RH) at the sensor head at the aforementioned same-plane orientation and observing no optical response, even at 6.0 LPM. Hence, any observed optical response are purely due to changes in the local RH.

In terms of flow-rate optimization to shorten the response time, it is revealed in Fig. S2 that to attain the shortest possible response time, a minimum threshold of 3.0 LPM must be met. This is because faster flow rates can: (a) ascertain a passage of near-pure RH, while slower flow rates allows more significant diffusion and mixing with the ambient air along the way to the sensor head; and (b) ensure the wavefront of moisture propagation is more or less uniform, such that the response time incurred at the light-water interaction stage is minimized.

The temperature dependence of hygrometers is more complex than that of other sensors. This is because changing the temperature not only affects the optical response of the sensor head, but also changes the RH. Both change the sensitivity: (a) temperature change, thermal expansion and thermo-optic refractive index changes, mode distribution change, optical response change, sensitivity change; and (b) temperature change, water-vapor density and dew-point change, RH change, sensitivity change. Hence, it should be noted that one set of calibration data is only valid for a specific operating temperature (e.g. room temperature of ~22 °C in this case) and pressure (e.g. typical atmospheric pressure of ~1015 hPa in this case). Temperature tests shown in Fig. S3 involving a hotplate, the humidity generator (i.e. maintains desired RH despite temperature changes) and the sensor heads reveals that the temperature-induced changes in the sensor head (part a) causes minor variations in the detected signal at a constant RH. To determine the appropriate calibration data to use in a real measurement environment, the hygrometer needs to be assisted by a thermometer.

Both hygrometers-under-test recover well after reaching dew point, taking under 3 s if imparted by a short human-breath, as shown in Fig. S4. Additionally, they show resilience to vibrations when handheld, judging by the negligible optical modulation.

## Conclusions

We have demonstrated a new class of humidity sensor that exhibits the shortest-ever response time. The sensor head comprises of an optical microfiber of 10 µm diameter and 2 mm length configured to form a compact U-shaped probe, and functionalized with a polyelectrolyte multilayer coating of 1.0 bilayer. Owing to the extremely fast water adsorption/desorption by the nanoporous polyelectrolyte multilayer coating, the response time is only 3 ms and the recovery time is 36 ms. The sensitivity is 0.02%/%RH (i.e. 0–72%RH), 0.4%/%RH (i.e. 72–80%RH), and 0.04%/%RH (i.e. 80–99%RH), while the detection limit is 32.6%RH (i.e. 0–72%RH), 1.6%RH (i.e. 72–80%RH) and 16.3%RH (i.e. 80–99%RH). The optical sensor head is impervious to electromagnetic interference due to its dielectric construction, has a strong evanescent field interacting with the polyelectrolyte multilayer coating, can be bent into a compact package, can probe confined spaces with its flexible fiber connections, and it is potentially low cost. For practicality, the sensor head can be packaged inside a rigid casing with a series of windows aligned with the direction of the fiber taper to ensure that any air flow would have a minimum impact on the geometrical stability of the sensor head.

## Experimental Section

### Optical fiber taper

The taper settings implemented by the fusion splicer (i.e. Fujikura FSM-100P+) consisted of: (a) a starting optical fiber of 125 μm diameter and 1 m length, down-taper length of 2 mm, uniform waist diameter of 10 μm, uniform waist length of 2 mm, up-taper length of 2 mm; (b) absolute power of 242 bits, relative power of −50 bits, waist add power of −12 bits; and (c) pull speed of ~0.1 mm/s, feed speed of 0.0143 mm/s.

### Polyelectrolyte coating

(PDDA/PSS)_N_ coatings were prepared on the on piranha-solution- hydroxylated optical microfibers, by alternating immersion of the substrates into the aqueous solutions of PDDA and PSS for 18 min until the desired layer number was reached. Each immersion step was followed by thorough rinsing with water and dried with N_2_ flow. The key to building up coating thickness is based on successive layers overcompensating the charge on the previous surface to reverse its charge for the adsorption of the next layer. PDDA and PSS aqueous solutions contain PDDA or PSS with concentrations of 1 mg/mL, and NaCl with a concentration of 1.0 M.

## Electronic supplementary material

Supplementary Information
