# Author Correction: Ultra-fast Hygrometer based on U-shaped Optical Microfiber with Nanoporous Polyelectrolyte Coating

**DOI:** 10.1038/s41598-018-22956-9

**Published:** 2018-03-22

**Authors:** George Y. Chen, Xuan Wu, Yvonne Qiongyue Kang, Li Yu, Tanya M. Monro, David G. Lancaster, Xiaokong Liu, Haolan Xu

**Affiliations:** 1 Laser Physics and Photonic Devices Laboratories, School of Engineering, University of South Australia, Mawson Lakes, South Australia 5095 Australia; 2 Future Industries Institute, University of South Australia, Mawson Lakes, South Australia 5095 Australia; 3 Shenzhen Key Laboratory of Laser Engineering, College of Optoelectronic Engineering, Shenzhen University, Shenzhen, 518060 China; 4 Key Laboratory of Optoelectronic Devices and Systems of Ministry of Education and Guangdong Province, College of Optoelectronic Engineering, Shenzhen University, Shenzhen, 518060 China

Correction to: *Scientific Reports* 10.1038/s41598-017-08562-1, published online 11 August 2017

The original version of this Article incorrectly listed Xuan Wu, and not Xiakong Liu, as a corresponding author. The correct corresponding authors for this Article are George Y. Chen, Xiaokong Liu and Haolan Xu. Correspondence and request for materials should be addressed to G.Y.C. (email: george.chen@unisa.edu.au) or X.L. (email: xiaokong.liu@unisa.edu.au) or H.X. (email: haolan.xu@unisa.edu.au). This error has now been corrected in the HTML and PDF versions of the Article.

