# Supplementary material for: Ultra-fast Hygrometer based on U-shaped Optical Microfiber with Nanoporous Polyelectrolyte Coating
Source: Sci Rep. 2017 Aug 11;7:7943. doi: 10.1038/s41598-017-08562-1 (PMC5554257; doi:10.1038/s41598-017-08562-1)
Supplement: Supplementary file 1 — Supplementary Information [file 41598_2017_8562_MOESM1_ESM.doc]

**Supplementary Information**

**Ultra-fast Hygrometer based on U-shaped Optical Microfiber with Nanoporous Polyelectrolyte Coating**

George Y. Chen,^1,*^ Xuan Wu,^2,*^ Yvonne Qiongyue Kang,^1^ Li Yu,^3,4^
Tanya M. Monro,^1^ David G. Lancaster,^1^ Xiaokong Liu,^2^ and Haolan Xu^2^

^1^ Laser Physics and Photonic Devices Laboratories, School of Engineering, University of South Australia, Mawson Lakes, South Australia 5095, Australia

^2^ Future Industries Institute, University of South Australia, Mawson Lakes, South Australia 5095, Australia

^3^ Shenzhen Key Laboratory of Laser Engineering, College of Optoelectronic Engineering, Shenzhen University, Shenzhen, 518060, China

^4^ Key Laboratory of Optoelectronic Devices and Systems of Ministry of Education and Guangdong Province, College of Optoelectronic Engineering, Shenzhen University, Shenzhen 518060, China

^*^ Denotes co-first authors

Address correspondence to G. Y. Chen, X. Liu and H. Xu.
Email: george.chen@unisa.edu.au
 xiaokong.liu@unisa.edu.au
 haolan.xu@unisa.edu.au

**Figure S1.** Scanning electron microscope image for: (a) uncoated fiber, (b) 1.0 bilayer coated fiber, and (c) 10.0 bilayer coated fiber. Magnified images of the 10.0 bilayer microfiber at (d) high magnification, and (e) low magnification.

**Figure S2.** Relationship between nitrogen-gas flow rate and response time.

**Figure S3.** Thermal characteristics of the two probes while an ambient relative humidity of 45%RH is maintained.

**Figure S4.** Temporal characteristics of reaching a human-breath-induced dew point followed by recovery.
